# Time Waits for No One: Longitudinal Study on the Effects of an Anti-Stigma Seminar on the Psychology Student Population

**DOI:** 10.3390/ijerph18105441

**Published:** 2021-05-19

**Authors:** Luca Pingani, Sara Evans-Lacko, Sandra Coriani, Silvia Ferrari, Maria Filosa, Gian Maria Galeazzi, Mattia Lorenzini, Tommaso Manari, Alessandro Musetti, Anna Maria Nasi, Christian Franceschini

**Affiliations:** 1 Department of Biomedical, Metabolic and Neural Sciences, Università degli Studi di Modena e Reggio Emilia, 41125 Modena, Italy; silvia.ferrari@unimore.it (S.F.); gianmaria.galeazzi@unimore.it (G.M.G.); 178289@studenti.unimore.it (M.L.); 2 Department of Health Professions, Azienda USL—IRCCS di Reggio Emilia, 42122 Reggio Emilia, Italy; sandra.coriani@ausl.re.it (S.C.); annamaria.nasi@ausl.re.it (A.M.N.); 3 Department of Mental Health, Azienda USL—IRCCS di Reggio Emilia, 42122 Reggio Emilia, Italy; 4 Care Policy Evaluation Centre, London School of Economics and Political Science, London WC2A 2AE, UK; s.evans-lacko@lse.ac.uk; 5 Centre for Global Mental Health, Institute of Psychiatry, Psychology and Neuroscience, King’s College London, London SE5 8AF, UK; 6 Department of Medicine and Surgery, University of Parma, 43125 Parma, Italy; filosa.maria@hotmail.it (M.F.); christian.franceschini@unipr.it (C.F.); 7 Department of Humanities, Social Sciences and Cultural Industries, University of Parma, 43121 Parma, Italy; tommaso.manari@unipr.it (T.M.); alessandro.musetti@unipr.it (A.M.)

**Keywords:** mental illness stigma, discrimination, stereotypes, social and political issues

## Abstract

The primary aim is to describe the changes in the knowledge of mental health conditions, the attitudes toward the mentally ill, and the intended behaviour towards people with mental illness among the entire student population of the third year of a degree course in Psychology. A total of 570 students attended a seminar on stigma towards mental illness and were invited to complete an online survey which collected data on sociodemographic characteristics and three validated questionnaires evaluating different aspects of stigma at three different time points (pre-intervention, post-intervention, and at one year follow up). A total of 253 students (44.39%) completed the questionnaires at t0, t1, and t2. The mean age of the sample was 23.7 (SD = ±5.89), and 86.96% (*n* = 220) were females. Between t0 and t1, a statistically significant improvement was observed for all three outcomes, while the intended behaviour outcome was no longer significant between t1 and t2 (Z = −0.70; *p* = 0.48). Females and who participated live at the seminar maintained a significant knowledge of mental illness and a better attitude toward community mental health care. The effects of the seminar focused on reducing stigma tended to diminish over time at one year follow-up, particular in relation to intended behaviour.

## 1. Introduction

In 1963, the American sociologist Goffman used the term “stigma” to indicate those attributes (ethnic, religious, physical, etc.) that connoted those who owned them as “... tainted, discounted one” [1]. Goffman’s first definition, thanks to the advances highlighted in the research, was further developed by Link and Phelan, who described the constituent components of the stigma process: labelling, stereotyping separation, status loss, and discrimination [2,3]. 

Lack of knowledge generates stereotypes that are assimilated and considered as truthful by the general population (mental health literacy). Agreement with the stereotype can result in a particular attitude, for example, fear of whether people with a mental illness are considered dangerous or capable of violent actions. The emotional experience ultimately leads to discriminatory behaviours, such as requiring that people with mental illness be locked up in institutions (asylums) that isolate them from society [4,5,6].

Besides the already mentioned public stigma, other types of stigma related to mental illness have been described in the literature: structural stigma, self-stigma, felt or perceived stigma, experienced stigma, label avoidance, courtesy stigma, and spiritual stigma [3,7,8]. 

The phenomenon of stigma towards mental illness generates dramatic consequences made evident by a number of studies. For example, stigma is the first obstacle to seeking help from mental health professionals [9,10,11,12], and it can lead from a decrease of autonomy and self-efficacy [13] to a worsening of the psychopathological condition and even to suicidal behaviours [14,15]: The World Health Organization (WHO) identified general stigma toward those with mental illness as the greatest barrier to effective psychiatric patient care [16]. Of all the consequences of stigma, those affecting social relations are the most dramatic: feelings of shame, social isolation, and difficulties with personal relationships. Finally, stigma is a barrier to achieving life goals, such as having a job, living alone independently, having a stable emotional relationship, or completing education [6,17,18,19,20]. 

Healthcare professionals and especially mental health workers can be both victims and perpetrators of stigmatizing attitudes. An interesting paper by Bhugra and colleagues [21] described, for example, how psychiatrists and psychiatric patients have always been stigmatized against. Several studies have shown that medical students do not believe that psychiatry is a medical discipline or that it is less scientific and precise than the others [22,23]. However, evidence in the literature also shows how mental health service users have experienced discriminatory attitudes from general practitioners and health professionals [24]. Nordt and colleagues showed that psychiatrists have more negative stereotypes than the general population and that there are no substantial differences between the social distance maintained by mental health professionals and the general population [6,25].

In order to define the presence and to fight stigmatizing attitudes in health professionals, several studies were conducted with the involvement of undergraduate students in health degree courses: psychology students [16,26,27], nursing students [28,29,30], medical students [31], and general mental health professional students [20]. 

The first aim of this longitudinal study was to describe the changes over time in the knowledge of mental disorders, the attitudes toward people who are mentally ill, and the intended behaviour towards people with mental illness in the entire student population of the third year of the degree course in Psychology of the Inter-University of Parma with Modena and Reggio Emilia after attending a Clinical Psychology course including a seminar on stigma towards mental illness. Secondly, we investigated whether changes in stigma outcomes differed for certain subpopulations: males and females, students who attended lessons in person vs. those who attended virtually, and students who had/did not have a first- or second-degree relative with a mental illness. 

Based on previous studies [32,33,34] that underlined how the evidence is not homogeneous about the effectiveness of educational strategies especially regarding the maintenance of the effect over time, we hypothesized that: the seminar would have an immediate positive impact (reduction of stigma in behaviour and attitude and increase of knowledge between t0 and t1) in the student population; however, the benefit would not remain stable over time, with a reduction between t1 and t2.

## 2. Materials and Methods

### 2.1. Sample and Intervention

For two consecutive academic years (A.Y.) (2018/2019 and 2019/2020), the students in the third year of the inter-university degree course in Psychology (University of Parma and University of Modena and Reggio Emilia) (*n* = 300 in 2018/2019; *n* = 270 in 2019/2020) were invited to complete an online survey that collected data on sociodemographic characteristics and validated stigma outcomes related to knowledge, attitudes, and intended stigmatising behaviour. 

Participants completed the survey at three different time points: two weeks prior to the start of the Clinical Psychology lectures; in the time window between 80 and 150 days after the end of the Clinical Psychology lectures; and in the time window between 240 and 330 days from the second compilation. The Clinical Psychology course is around 64 h and includes a 4-h seminar on stigma towards mental illness (including information on definitions and classifications, strategies to fight stigma, psychometric tools, and international campaigns against stigma). Our sample size calculation suggested that we needed 230 participants to complete the study based on a total population of 570 students and considering 5% as margin of error (confidence level: 95%).

### 2.2. Sociodemographic Information and Stigma Questionnaire

Students who agreed to fill in the questionnaires were asked for the following socio-demographic information: gender, age, class participation (face to face or remotely), having already participated in stigma awareness events, and having first- and second-degree relatives who have/had a mental illness.

The online survey comprised three validated psychometric questionnaires. The Italian version of the Mental Health Knowledge Schedule (MAKS-I) [35,36] is a self-administered questionnaire composed of 12 items scored on a Likert scale (from 1: “Strongly Disagree” to 5: “Strongly Agree”). “Don’t know” is coded as neutral (value of 3) according to the scoring guidelines. The MAKS-I questionnaire is categorised into two parts. The first six statements can be summed into a total score representing stigma-related mental health knowledge (the higher the score, the greater the knowledge of mental illness). Items from 7 to 12 assess recognition and familiarity with six different conditions.

The CAMI Scale (community attitudes to mentally illness) refers to attitudes towards people who are mentally ill. Participants rate the 27 statements from 1, “Strongly Disagree”, to 5, “Strongly Agree”, and a high score corresponds with positive attitude [37,38,39]. 

The Italian version of the Reported and Intended Behaviour Scale (RIBS) is a self-administered questionnaire composed of eight items evaluating reported and intended behaviours across four different domains: (1) living with, (2) working with, (3) living nearby, and (4) continuing a relationship with someone with a mental health problem. The total intended behaviour score is calculated by summing the answers for items five through eight. A higher score indicates a higher level of intended behaviour and/or contact with someone with a mental health problem [40,41]. 

### 2.3. Statistical Analysis

The interval variables were described using means and standard deviations (SDs), and the categorical and ordinal variables were described using frequencies and percentages. Homogeneity test between students of the two A.Y. were carried out through Mann–Whitney U test for interval variables and chi-square test for ordinal, categorial, and nominal variables [42]. 

The Shapiro–Wilk Test was used to verify if the scores obtained on the questionnaires have a normal distribution [43]. To verify the change over time of the scores obtained in the three questionnaires, the Friedman test as a non-parametric statistical test was used [44]. The comparison in the sub-populations was carried out using the Wilcoxon test [45].

## 3. Results

Of the 570 students contacted, 302 (52.98%) agreed to be involved in the study, and 253 (44.39%) completed the questionnaires at t0, t1, and t2. The mean age of the sample was 23.74 (SD = ±5.89), and 86.96% (*n* = 220) was female. Only 40 students (15.81%) had already participated in stigma awareness events, while 25.30% (64) have/had a first- or second-degree relative with a psychiatric disorder (*n* = 64; 25.30%). The homogeneity test failed only for the age variable (24.78 ± 6.18 vs. 22.94 ± 5.55; *p* < 0.001). The sociodemographic characteristics of the sample and the test of homogeneity are described in Table 1.

Due to the high difference in the representation of males vs. females, it was considered appropriate to run the analysis of subgroups as a sensitivity analysis.

Table 2 describes changes in stigma-related mental health knowledge (MAKS-I), community attitudes to mental illness (CAMI), and the intended behaviour towards people with mental illness (RIBS-I) at pre (t0), post (t1), and one year follow-up (t2) time points. Between t0 and t1, a statistically significant improvement was observed for all three outcomes. There was no significant change between t1 and t2 in intended behaviour (Z = −0.70; *p* = 0.48): the percentage of those who obtained a higher score and those who obtained the same score with respect to t0 are almost the same (*n* = 89; 35.89% vs. *n* = 87; 35.08%). Through the Friedman test, a statistically significant improvement was highlighted for all questionnaire scores in repeated measurements at t0, t1, and t2. (MAKS-I: Χ^2^ = 85.46; df = 2; *p* < 0.001—CAMI: Χ^2^ = 195.59; df = 2; *p* < 0.001—RIBS-I: Χ^2^ = 83.99; df = 2; *p* < 0.001).

Both the male and female subpopulations (Table 3) had a statistically significant improvement between t0 and t1. From the comparison between t1 and t2, for the male subpopulation, an improvement emerged for the three questionnaires, but it was not statistically significant. For the female subpopulation, there was a statistically significant improvement only for stigma-related mental health knowledge (MAKS-I) (Z = −2.16; *p* < 0.001) and attitudes (CAMI) (Z = −3.10; *p* = 0.002). For both subpopulations, there was a statistically significant improvement for all questionnaires considering the repeated measures at t0, t1, and t2. 

Both students attending face-to-face lectures in the classroom and those attending virtual lectures had a statistically significant improvement between t0 and t1 in all three stigma outcomes. There was no statistically significant improvement between t1 and t2 among those attending in person or virtually and regarding intended behaviour towards people with mental illness (Classroom: Z = −0.72, *p* = 0.47; Distance learning: Z = −0.14, *p* = 0.89). Students who attended the remote frequency (RF) course did not experience a statistically significant improvement between t1 and t2 in the stigma-related knowledge of mental disorders (Z = −1.29; *p* = 0.20) or attitudes (Z = −0.37; *p* = 0.71). Trends over time of MAKS-I, CAMI, and RIBS-I for students who attended in the classroom and those in distance learning are described in Table 4.

A statistically significant improvement from t0 to t1 was observed both among those who have/had a first- or second-degree relative with a psychiatric disorder and among those who did not. Between t1 and t2, there was only a statistically significant improvement in attitudes among those who have/do not have a relative with a psychiatric disorder. For both subpopulations, there was a statistically significant improvement for all stigma outcomes (knowledge, attitude, and behaviour) considering the repeated measures at t0, t1, and t2.

## 4. Discussion

Before proceeding with the discussion of the results obtained, we describe the limitations that characterize this study. First, the homogeneity test failed by comparing the gender of the student population of the two academic years (A.Y. 2018/2019 and 2019/2020): the percentage of female students is slightly higher in the A.Y. 2019/2020 (89.51% vs. 92.64%). Furthermore, the number of males and females is very different; therefore, the results obtained must be compared with those in the literature with caution. The questionnaires validated in the Italian language used in this study provided decidedly good psychometric qualities in the previous validation processes [36,41]; however, it is necessary to take their results carefully as there are still few studies regarding their reliability and validity. Finally, the number of students who completed the study is sufficient (253 compared to the 230 requested), but a response rate of 44.39% can hide some critical issues: probably the most motivated students participated in the study, while the data relative to the less interested or more stigmatizing ones, we could hypothesize, did not appear in the study. Despite the limitations, we believe that there are results worthy of attention and discussion.

We found that, for the entire students’ populations, intended stigmatising behaviour improved statistically significantly only between t0 and t1. Several studies in the literature have shown that non-stigmatizing effects on behaviour of anti-stigma interventions reduce over time: Evans-Lacko and colleagues, evaluating a brief anti-stigma campaign (pre, during, and post), described how there were not evident improvements for attitudinal or behavioural elements [46]. Even in anti-stigma campaigns (e.g., “Time to Change”), it has been verified that, despite the efforts made, there was no significant improvement in behaviour among the general public population [47]. 

In the results of the trend over time in subpopulations (male vs. female, Table 3; classroom vs. distance learning, Table 4; having or not a first- or second-degree relative with a psychiatric disorder, Table 5), it emerges with even greater clarity that the effectiveness of the anti-stigma activity tends to decrease over time: all the evaluations between t0 and t1 are positive and statistically significant; in the comparison between t1 and t2, change is always positive (less stigma) but rarely statistically significant. We can hypothesize that, over time, the anti-stigma activity loses its effectiveness, and we could therefore speak of a dose effect: the anti-stigma activity leads to short-term benefits, but its effect tends to fade over time as already described in the literature [48].

By analysing the results over time in the different subpopulations, it appears that the female population, unlike the male population, maintains a significant knowledge of mental illness and a better attitude toward people who are mentally ill. This finding may have been affected by the differential representation of female vs. male subjects in the study population. The results obtained from this study are congruent with literature: females tend to have greater knowledge of mental illness [12,49,50] and more positive attitudes [15]. Studies on populations of university students also seem to confirm this: Rafal and colleagues [51], for example, described how college men are less likely to correctly identify depression, anxiety, and severe stress. Another study described how male psychology students have a higher level of stigmatizing attitudes than the female population [26].

As described in the systematic review conducted by Janoušková and colleagues [52], anti-stigma strategies carried out using video interventions (e.g., an interview with a person with mental illness in recovery) showed improvements in stigmatising attitudes and appeared to be more effective than other interventions, such as classical face-to-face educational sessions or simulation of hallucinations. In particular, the effectiveness of video-based contact interventions have been shown to be particularly effective: adding a video-based contact can significantly improve program effectiveness when presented following a lesson; short video contact intervention also reduced stigma in a sample of nursing students [53]. In our study, the difference between attending the stigma seminar in the classroom or seeing it recorded online was described. Our data seem to favour the presence in classroom because, comparing second and third assessment (t1–t2), there was a non-significant improvement (the percentage of participants who obtained a score at t2 higher than that obtained at t1 is always greater than that of those who obtained a lower score) in all three questionnaires in the virtual group while, for the students in the classroom, a statistically significant improvement remained for knowledge of mental disorders and attitudes toward people who are mentally ill. Our results, therefore, seem not to confirm what is described in literature: for example, Clements and colleagues [54] described that DVD and live interventions were equally effective and interventions with social contact (DVD/live) were more effective than the lecture alone (which also does not provide for social contact). We can hypothesize that the difference can be that the recorded lesson was not designed for this purpose but only to allow the working students to attend the event.

In the literature, there are several studies that demonstrate that contact is the preferred strategy in the fight against stigma [6,55]. As described by Pettigrew and Tropp [56], intergroup contact typically reduces intergroup prejudice: the meta-analysis results suggest that greater intergroup contact is generally associated with lower levels of prejudice. The literature is more mixed about the association between having a family member with a mental illness and stigmatizing attitudes. For example, Gonzales-Sanguino and colleagues [16] highlighted how greater implicit stigma was found in people who had a family member with a psychiatric diagnosis, while Korszun and colleagues [31] described that fewer stigmatizing attitudes are associated with personal experience of mental health treatment or that among family and friends. In a previous study that considered a psychology student population [35], we found that there was no association between having a family member with a mental health problem and knowledge of different clinical conditions. In the population of psychology students of this study, minimal differences in t0 scores were found between the different subpopulations (having/not having a family member with a psychiatric disorder) for MAKS-I and RIBS-I. Even between t1 and t2, only those who had no family member with a mental illness showed a statistically significant improvement. 

## 5. Conclusions

The fight against stigma is undoubtedly one of the priority goals of mental health. In this study that involved psychology students, despite the limitations described, it was possible to identify the positive effects of a seminar on stigma, but these tended to reduce over time. Furthermore, it is emphasized that interventions must always be evaluated in the respective subpopulations to have more reliable and useful results. In order to overcome the problem of the reduction over time of sensitivity to the theme of stigma following anti-stigma initiatives, it is important to undertake new research aiming to evaluate activities that can be repeated over time (annual seminars) with an increasing involvement of peer-workers and with increasing accessibility (web, social networks, and distance learning).

## Figures and Tables

**Table 1:** Sociodemographic characteristics of the sample and verification of the homogeneity.

	**Mean ± SD**			**Homogeneity**
	Total sample	Academic Year 2018/2019	Academic Year 2019/2020	Mann–Whitney U test
Age	23.74 ± 5.89	24.78 ± 6.18	22.94 ± 5.55	*p* < 0.001
	***n* (%)**			**Homogeneity**
	Total sample	Academic Year 2018/2019	Academic Year 2019/2020	Χ^2^ test
Sex				
Male	33 (13.04%)	18 (16.36%)	15 (10.49%)	Χ^2^ = 1.89; df = 1; *p* = 0.17
Female	220 (86.96%)	92 (83.64%)	128 (89.51%)	
Participation in lectures				
In the classroom	172 (67.98%)	69 (62.73%)	103 (72.03%)	Χ^2^ = 2.47; df = 1; *p* = 0.12
In distance learning	81 (32.02%)	41 (37.27%)	40 (27.97%)	
Participation in mental health stigma events				
Yes	40 (15.81%)	19 (17.27%)	21 (14.69%)	Χ^2^ = 0.31; df = 1; *p* = 0.58
No	213 (84.19%)	91 (82.73%)	122 (85.31%)	
Having a first- or second-degree relative with a psychiatric disorder				
Yes	64 (25.30%)	29 (26.36%)	35 (24.48%)	Χ^2^ = 0.12; df = 1; *p* = 0.73
No	189 (74.70%)	81 (73.64%)	108 (75.52%)	

**Table 2:** Trend over time of the scores obtained in the knowledge of mental disorders (MAKS-I), the attitudes towards people who are mentally ill (CAMI), and the intended behaviour towards people with mental illness (RIBS-I).

	t0 (Means ± SD)	t1 (Means ± SD)	t2 (Means ± SD)	Wilcoxon Test (t0–t1)		Wilcoxon Test (t1–t2)		Friedman Test (t0–t1–t2)				
									IQ ^a^	IIQ ^b^	IIIQ ^c^	
MAKS-I (*n* = 247)	20.85 ± 2.66	22.26 ± 2.54	22.69 ± 2.58	t1 < t0: 56 (22.67%)	Z = −7.83; *p* < 0.001	t2 < t1: 89 (36.03%)	Z = −2.56; *p* = 0.01	t0	19	21	22	Χ^2^ = 85.46; df = 2; *p* < 0.001
				t1 > t0: 157 (63.56%)		t2 > t1: 120 (48.58%)		t1	20	22	24	
				t1 = t0: 34 (13.77%)		t2 = t1: 38 (15.38%)		t2	21	23	24	
CAMI (*n* = 248)	109.08 ± 2.58	115.87 ± 8.21	116.89 ± 8.97	t1 < t0: 35 (14.11%)	Z = −11.26; *p* < 0.001	t2 < t1: 90 (36.29%)	Z = −3.34; *p* = 0.001	t0	103	110	115	Χ^2^ = 195.59; df = 2; *p* < 0.001
				t1 > t0: 204 (82.56%)		t2 > t1: 142 (57.26%)		t1	112	116.50	122	
				t1 = t0: 9 (3.63%)		t2 = t1: 16 (6.45%)		t2	112	118.50	123	
RIBS-I (*n* = 248)	15.98 ± 2.58	17.35 ± 2.28	17.40 ± 2.36	t1 < t0: 50 (20.16%)	Z = −8.53; *p* < 0.001	t2 < t1: 72 (29.03%)	Z = −0.70; *p* = 0.48	t0	14.25	16	18	Χ^2^ = 83.99; df = 2; *p* < 0.001
				t1 > t0: 144 (58.06%)		t2 > t1: 89 (35.89%)		t1	16	18	19	
				t1 = t0: 54 (21.77%)		t2 = t1: 87 (35.08%)		t2	16	18	19	

a: first quartile; b: second quartile; c: third quartile.

**Table 3:** Trend over time of the scores obtained in the knowledge of mental disorders (MAKS-I), the attitudes towards people who are mentally ill (CAMI), and the intended behaviour towards people with mental illness (RIBS-I) for male and female subpopulations.

	Male/Female	t0 (Means ± SD)	t1 (Means ± SD)	t2 (Means ± SD)	Wilcoxon Test (t0–t1)		Wilcoxon Test (t1–t2)		Friedman Test (t0–t1–t2)				
										IQ ^a^	IIQ ^b^	IIIQ ^c^	
MAKS-I	M (*n* = 31)	21.26 ± 3.44	22.90 ± 2.33	23.65 ± 2.58	t1 < t0: 7 (22.58%)	Z = −3.01; *p* = 0.003	t2 < t1: 9 (29.03%)	Z = −1.56; *p* = 0.12	t0	19	21	24	Χ^2^ = 10.77; df = 2; *p* = 0.005
					t1 > t0: 18 (58.06%)		t2 > t1: 15 (48.39%)		t1	21	23	25	
					t1 = t0: 6 (19.35%)		t2 = t1: 7 (22.58%)		t2	22	24	25	
	F (*n* = 216)	20.79 ± 2.53	22.17 ± 2.56	22.56 ± 2.56	t1 < t0: 49 (22.69%)	Z = −7.23; *p* < 0.001	t2 < t1: 80 (37.04%)	Z = −2.16; *p* = 0.03	t0	19	21	22	Χ^2^ = 74.91; df = 2; *p* < 0.001
					t1 > t0: 139 (64.35%)		t2 > t1: 105 (48.61%)		t1	20	22	24	
					t1 = t0: 28 (12.96%)		t2 = t1: 31 (14.35%)		t2	21	23	24	
CAMI	M (*n* = 32)	109.75 ± 9.82	117.09 ± 7.30	117.97 ± 8.49	t1 < t0: 5 (15.63%)	Z = −4.17; *p* < 0.001	t2 < t1: 11 (34.38%)	Z = −1.25; *p* = 0.21	t0	104	110.50	117.75	Χ^2^ = 26.80; df = 2; *p* < 0.001
					t1 > t0: 25 (78.13%)		t2 > t1: 21 (65.63%)		t1	112	118	122	
					t1 = t0: 2 (6.25%)		t2 = t1: 0		t2	114	121	124.75	
	F (*n* = 216)	108.98 ± 8.32	115.69 ± 8.33	116.73 ± 9.05	t1 < t0: 30 (13.89%)	Z = −10.45; *p* < 0.001	t2 < t1: 79 (36.57%)	Z = −3.10; *p* = 0.002	t0	103	110	115	Χ^2^ = 169.26; df = 2; *p* < 0.001
					t1 > t0: 179 (82.87%)		t2 > t1: 121 (56.02%)		t1	112	116	122	
					t1 = t0: 7 (3.24%)		t2 = t1: 16 (7.41%)		t2	112	118	123	
RIBS-I	M (*n* = 33)	16.30 ± 2.28	17.58 ± 2.11	17.48 ± 2.15	t1 < t0: 3 (9.09%)	Z = −3.51; *p* < 0.001	t2 < t1: 7 (21.21%)	Z = −0.09; *p* = 0.93	t0	14.50	16	18	Χ^2^ = 18.23; df = 2; *p* < 0.001
					t1 > t0: 18 (54.55%)		t2 > t1: 11 (33.33%)		t1	16	18	19.50	
					t1 = t0: 12 (36.36%)		t2 = t1: 15 (45.45%)		t2	16	17	20	
	F (*n* = 215)	15.93 ± 2.63	17.31 ± 2.31	17.39 ± 2.40	t1 < t0: 47 (21.86%)	Z = −7.79; *p* < 0.001	t2 < t1: 65 (30.23%)	Z = −0.81; *p* = 0.42	t0	14	16	18	Χ^2^ = 67.39; df = 2; *p* < 0.001
					t1 > t0: 126		t2 > t1: 78 (36.28%)		t1	16	18	19	
					t1 = t0: 42 (19.53%)		t2 = t1: 15 (6.98%)		t2	16	18	19	

a: first quartile; b: second quartile; c: third quartile.

**Table 4:** Trend over time of the scores obtained in the knowledge of mental disorders (MAKS-I), the attitudes towards people who are mentally ill (CAMI), and the intended behaviour towards people with mental illness (RIBS-I) for students who attended in the classroom and those in distance learning.

	Classroom/Distance Learning	t0 (Means ± SD)	t1 (Means ± SD)	t2 (Means ± SD)	Wilcoxon Test (t0–t1)		Wilcoxon Test (t1–t2)		Friedman Test (t0–t1–t2)				
										IQ ^a^	IIQ ^b^	IIIQ ^c^	
MAKS-I	Classroom (*n* = 169)	20.64 ± 2.51	22.11 ± 2.51	22.55 ± 2.39	t1 < t0: 33 (19.53%)	Z = −7.01; *p* < 0.001	t2 < t1: 60 (35.50%)	Z = −2.22; *p* = 0.03	t0	19	21	22	Χ^2^ = 66.45; df = 2; *p* < 0.001
					t1 > t0: 107 (63.31%)		t2 > t1: 84 (49.70%)		t1	20	22	24	
					t1 = t0: 29 (17.16%)		t2 = t1: 25 (14.79%)		t2	21	23	24	
	Distance learning (*n* = 78)	21.31 ± 2.91	22.60 ± 2.59	23 ± 2.95	t1 < t0: 23 (29.49%)	Z = −3.71; *p* < 0.001	t2 < t1: 29 (37.18%)	Z = −1.29; *p* = 0.20	t0	19	21	23	Χ^2^ = 19.86; df = 2; *p* < 0.001
					t1 > t0: 50 (74.10%)		t2 > t1: 36 (46.15%)		t1	20.75	22	25	
					t1 = t0: 5 (6.41%)		t2 = t1: 13 (16.67%)		t2	21	23	25.25	
CAMI	Classroom (*n* = 170)	108.97 ± 8.53	116.05 ± 7.84	117.61 ± 8.64	t1 < t0: 23 (13.53%)	Z = −9.43; *p* < 0.001	t2 < t1: 56 (32.94%)	Z = −3.73; *p* < 0.001	t0	103	110.50	115	Χ^2^ = 153.63; df = 2; *p* < 0.001
					t1 > t0: 141 (82.94%)		t2 > t1: 102 (60%)		t1	111	117	122	
					t1 = t0: 6 (3.53%)		t2 = t1: 12 (7.06%)		t2	113.75	119	124	
	Distance learning (*n* = 78)	109.32 ± 8.52	115.50 ± 9.01	115.32 ± 9.53	t1 < t0: 12 (15.38%)	Z = −6.14; *p* < 0.001	t2 < t1: 34 (43.59%)	Z = −0.37; *p* = 0.71	t0	104	110	115	Χ^2^ = 45.53; df = 2; *p* < 0.001
					t1 > t0: 63 (80.77%)		t2 > t1: 40 (51.28%)		t1	113	116	121	
					t1 = t0: 3 (3.85%)		t2 = t1: 4 (5.13%)		t2	111	117	122	
RIBS-I	Classroom (*n* = 169)	16.09 ± 2.41	17.46 ± 2.12	17.56 ± 2.50	t1 < t0: 33 (19.53%)	Z = −7.23; *p* < 0.001	t2 < t1: 47 (27.81%)	Z = −0.72; *p* = 0.47	t0	15	16	18	Χ^2^ = 59.47; df = 2; *p* < 0.001
					t1 > t0: 99 (58.58%)		t2 > t1: 66 (39.05%)		t1	16	18	19	
					t1 = t0: 37 (21.89%)		t2 = t1: 56 (33.14%)		t2	16	18	20	
	Distance learning (*n* = 79)	15.76 ± 2.92	17.11 ± 2.60	17.06 ± 2.58	t1 < t0: 17 (21.52%)	Z = −4.59; *p* < 0.001	t2 < t1: 25 (31.65%)	Z = −0.14; *p* = 0.89	t0	14	16	18	Χ^2^ = 25.01; df = 2; *p* < 0.001
					t1 > t0: 45 (56.96%)		t2 > t1: 23 (29.11%)		t1	15	18	19	
					t1 = t0: 17 (21.52%)		t2 = t1: 31 (39.24%)		t2	16	17	19	

a: first quartile; b: second quartile; c: third quartile.

**Table 5:** Trend over time of the scores obtained in the knowledge of mental disorders (MAKS-I), the attitudes towards people who are mentally ill (CAMI), and the intended behaviour towards people with mental illness (RIBS-I) for students who have/not have a first- or second-degree relative with a psychiatric disorder.

	Having a First- or Second-Degree Relative with a Psychiatric Disorder	t0 (Means ± SD)	t1 (Means ± SD)	t2 (Means ± SD)	Wilcoxon Test (t0–t1)		Wilcoxon Test (t1–t2)		Friedman Test (t0–t1–t2)				
										IQ ^a^	IIQ ^b^	IIIQ ^c^	
MAKS-I	Yes (*n* = 64)	21.52 ± 2.58	22.78 ± 2.45	23.45 ± 2.45	t1 < t0: 11 (17.19%)	Z = −3.68; *p* < 0.001	t2 < t1: 23 (35.94%)	Z = −1.77; *p* = 0.08	t0	20	21	23	Χ^2^ = 27.59; df = 2; *p* < 0.001
					t1 > t0: 41 (64.06%)		t2 > t1: 35 (54.59%)		t1	21	23	24	
					t1 = t0: 12 (18.75%)		t2 = t1: 6 (9.38%)		t2	22	24	25.75	
	No (*n* = 183)	20.62 ± 2.65	22.08 ± 2.56	22.43 ± 2.57	t1 < t0: 45 (24.59%)	Z = −6.86; *p* < 0.001	t2 < t1: 66 (36.07%)	Z = −1.90; *p* = 0.06	t0	19	21	22	Χ^2^ = 58.33; df = 2; *p* < 0.001
					t1 > t0: 116 (63.39%)		t2 > t1: 85 (49.13%)		t1	20	22	24	
					t1 = t0: 22 (12.02%)		t2 = t1: 32 (17.49%)		t2	21	23	24	
CAMI	Yes (*n* = 61)	109.74 ± 7.71	117.339 ± 7.91	117.66 ± 7.70	t1 < t0: 7 (11.48%)	Z = −5.93; *p* < 0.001	t2 < t1: 28 (45.90%)	Z = −0.47; *p* = 0.64	t0	103.50	111	115.50	Χ^2^ = 51.30; df = 2; *p* < 0.001
					t1 > t0: 52 (85.25%)		t2 > t1: 30 (49.18%)		t1	112	119	123.50	
					t1 = t0: 2 (3.28%)		t2 = t1: 3 (4.92%)		t2	113.50	118	123	
	No (*n* = 187)	108.87 ± 8.77	115.38 ± 8.26	116.64 ± 9.36	t1 < t0: 28 (14.97%)	Z = −9.60; *p* < 0.001	t2 < t1: 62 (33.16%)	Z = −3.61; *p* < 0.001	t0	103	110	115	Χ^2^ = 146.06; df = 2; *p* < 0.001
					t1 > t0: 152 (81.28%)		t2 > t1: 112 (59.89%)		t1	111	116	121	
					t1 = t0: 7 (3.74%)		t2 = t1: 13 (6.95%)		t2	112	119	123	
RIBS-I	Yes (*n* = 62)	15.89 ± 2.93	17.35 ± 2.38	17.27 ± 2.32	t1 < t0: 11 (17.74%)	Z = −4.37; *p* < 0.001	t2 < t1: 19 (30.65%)	Z = −0.86; *p* = 0.39	t0	14	16	18	Χ^2^ = 18.43; df = 2; *p* < 0.001
					t1 > t0: 33 (53.23%)		t2 > t1: 22 (35.38%)		t1	15.75	18	19	
					t1 = t0: 18 (29.03%)		t2 = t1: 21 (33.87%)		t2	15.75	18	19	
	No (*n* = 186)	16.02 ± 2.46	17.43 ± 2.56	17.44 ± 2.38	t1 < t0: 39 (20.97%)	Z = −7.32; *p* < 0.001	t2 < t1: 53 (28.49%)	Z = −0.08; *p* = 0.94	t0	15	16	18	Χ^2^ = 66.62; df = 2; *p* < 0.001
					t1 > t0: 111 (59.68%)		t2 > t1: 67 (36.02%)		t1	16	18	19.25	
					t1 = t0: 36 (19.35%)		t2 = t1: 66 (35.48%)		t2	16	18	20	

a: first quartile; b: second quartile; c: third quartile.

## Data Availability

All data used for this study are available upon request by the corresponding author.
